# A Novel Method of Temporomandibular Joint Hypermobility Diagnosis Based on Signal Analysis

**DOI:** 10.3390/jcm10215145

**Published:** 2021-11-02

**Authors:** Justyna Grochala, Dominik Grochala, Marcin Kajor, Joanna Iwaniec, Jolanta E. Loster, Marek Iwaniec

**Affiliations:** 1 Department of Prosthodontics, Institute of Dentistry, Jagiellonian University Medical College, Jagiellonian University, 31-155 Kraków, Poland; justyna.lemejda@doctoral.uj.edu.pl (J.G.); jolanta.loster@uj.edu.pl (J.E.L.); 2 Institute of Electronics, AGH University of Science and Technology, 30-059 Kraków, Poland; 3 Faculty of Electrical Engineering, Automatics, Computer Science and Biomedical Engineering, AGH University of Science and Technology, 30-059 Kraków, Poland; mkajor@agh.edu.pl; 4 Department of Robotics and Mechatronics, Faculty of Mechanical Engineering and Robotics, AGH University of Science and Technology, 30-059 Kraków, Poland; joanna.iwaniec@agh.edu.pl; 5 Department of Process Control, Faculty of Mechanical Engineering and Robotics, AGH University of Science and Technology, 30-059 Kraków, Poland; iwaniec@agh.edu.pl

**Keywords:** hypermobile temporomandibular joint movement, auscultation, stethoscope, signal analysis, RDC/TMD

## Abstract

Despite the temporomandibular joint (TMJ) being a well-known anatomical structure its diagnosis may become difficult because physiological sounds accompanying joint movement can falsely indicate pathological symptoms. One example of such a situation is temporomandibular joint hypermobility (TMJH), which still requires comprehensive study. The commonly used official research diagnostic criteria for temporomandibular disorders (RDC/TMD) does not support the recognition of TMJH. Therefore, in this paper the authors propose a novel diagnostic method of TMJH based on the digital time–frequency analysis of sounds generated by TMJ. Forty-seven volunteers were diagnosed using the RDC/TMD questionnaire and auscultated with the Littmann 3200 electronic stethoscope on both sides of the head simultaneously. Recorded TMJ sounds were transferred to the computer via Bluetooth^®^ for numerical analysis. The representation of the signals in the time–frequency domain was computed with the use of the Python Numpy and Matplotlib libraries and short-time Fourier transform. The research reveals characteristic time–frequency features in acoustic signals which can be used to detect TMJH. It is also proved that TMJH is a rare disorder; however, its prevalence at the level of around 4% is still significant.

## 1. Introduction

A healthy temporomandibular joint (TMJ) is painless, hassle-free during chewing, speaking and eating and is soundless. Joint sounds play an important role in the examination, diagnosis and future treatment of temporomandibular disorders. There is a wide spectrum of sounds from joints. The internal disarrangement of TMJ leads to clinical symptoms, such as clicking, popping and crepitations. Another type of sound, called a “thud”, is identifiable during TMJ movements and is associated with hypermobile temporomandibular joint (TMJH). This may be considered as a disorder or a physiological condition.

TMJH is a state in which the correct disc position is observed but the maximal mouth opening leads to subluxation or luxation of the joint, which is accompanied by pain. Subluxation, or hypermobility, is the extended movement of TMJ during wide mouth opening. The correct anatomy of TMJ is associated with a smooth condylar head movement down into the top of the articular tubercle. In the group of patients, a temporary stop occurs during the widest opening of the mouth, due to a sudden, rapid transition in the joint. Such a stop results in a specific sound—clinically audible as a thud. The phenomenon is physiological and results from the specific anatomy of the articular tubercle, which can be caused by the excessive mouth opening during yawning, singing, vomiting, eating or through factors such as generalised joint hypermobility (GJH), Ehler Danlos syndrome, Marfan syndrome or intubation under general anaesthesia [1,2]. 

TMJH may also be considered as a non-physiological condition if a patient report chewing muscle disorders as well as TMJ pain and discomfort. The sound that appears in the final stage of opening of the mouth may be a sign of subluxation of the condyle, which moves forward [3]. Repeated episodes of subluxation may result in lengthening of the patient’s ligaments, potentially leading to disorders of the disc [4]. TMJH may also occur as a result of an injury or damage to the joint capsule. Some of the patients suffering from TMJH also have hypermobility of other joints in the body, which is known as generalised joint hypermobility (GJH). GJH results in an above-average range of motion in many joints. As there is no single test to diagnose GJH [5], various tests are commonly used, as reported by Carter and Wilkinson [6], Kirk et al. [7], Beighton and Horan [8], and Beighton et al. [9]. 

Pasinato et al. proved that GJH is a predisposing factor for the development of temporomandibular disorders (TMD). Of patients with TMD, 64.71% also had GJH, including TMJH [10].

Nosouhian et al. reported that TMJH occurs much more frequently in women (78.2%) than in men [11]. Moreover, Mehndiratta et al., using magnetic resonance imaging (MRI), noticed that TMJH is connected with an anatomic variant where the articular eminence has a steep and short posterior slope and a longer anterior slope [2].

Researchers and clinicians are currently working on novel methods of diagnosis, based on the adopted and modified physical methods, such as auscultation, X-rays, computed tomography (CT), ultrasonography, and MRI. However, non-invasive methods are preferred, since they are more convenient for the patients and can be performed without sophisticated tools and specialists. Therefore, the main goal of this research was to develop such a method based on the time–frequency acoustic signal representation.

In the well-known and commonly used RDC/TMD questionnaire, there is no diagnosis for patients with a sound like a thud, but additional medical imaging procedures, for example, can confirm the diagnosis of TMJH. The newest version of RDC/TMD, called diagnostic criteria for temporomandibular disorders (DC/TMD), provides the classification of this kind of sound. Unfortunately, the questionnaire is not yet available everywhere as it requires a reverse translation and the acceptance of the authors for general use, as is the case with the Polish version.

At present, the stethoscope is the standard instrument for the acoustic examination of TMJ [12,13,14]. Another option is the use of a more advanced device, for example the one created by Radke et al. They invented a system called joint vibration analysis (JVA) for diagnosing joint dysfunctions. The mechanism is based on sensing/measuring joint vibrations and analysing the intensity and frequency of the emitted vibrations [15].

The simplest commonly used and non-invasive diagnostic method is auscultation using stethoscopes. Such instruments enable the digital filtering of noise and redundant frequencies, and also support the acquisition of recorded signals for further analysis. Due to the development of this method, the problem of TMD detection can be solved by using electronic stethoscopes with an appropriate synchronisation module and dedicated software. Such a diagnostic system was unavailable a few years ago and the diagnosis was based solely on the experience of a dentist.

This approach also allows us to incorporate digital signal processing and implement dedicated algorithms supporting the detection of pathological syndromes and pattern recognition [16]. Therefore, seeking the patterns occurring in the signal, which correspond with real pathologies detected by a physician, is usually crucial in the development of algorithms facilitating an automated diagnosis.

For dentists, there is a problem with the diagnosis of TMJ disorders, so the solution with computer analysis seems to make it easier and more available for dentists during their routine daily work. The auscultation method is a basic procedure and is easy to use in the average dental clinic, but proper interpretation of the heard signals requires extensive experience in this particular field. To check non-objective, individual diagnosis, the physician can easily share patient recordings and consult with a specialist using the digital signals obtained with the proposed setup. More importantly, our system, apart from the quite obvious consultation capability, in a further, final version, will allow for direct interpretation as healthy, with pathologies or caused by hypermobility. In this form, it will be a direct and clear suggestion to the doctor about the type of sound and, consequently, the diagnosis. It will be a solution that gives an automatic result immediately after the examination, based on a non-invasive and cheap diagnostic procedure. Noticing that the signals are pathological would allow the patient to be more quickly referred to a specialist. This would improve patients’ quality of life through direct referral for appropriate treatment. It is now common that the recommendation for a dental consultation from a specialist of temporomandibular joint disorders is a final step, usually a very long time after the symptoms first appear.

Computer-aided auscultation also enables the visualisation of measurements, which significantly improves the effectiveness of teaching diagnosis based on auscultation of the patient. Research conducted at the University Hospital in Strasbourg shows that thanks to the sound visualisation tools, the percentage of correctly made auscultatory diagnoses among medical students increased from 64% to 80% [17]. The standard auscultatory examination of TMJ makes it difficult to distinguish between TMD, such as clicks, and TMJH, because of the similar audible effects accompanying both ailments. Such a diagnosis is also dependent on dentist’s experience and might be biased. The signal analysis proposed in this paper takes advantage of spectrogram which allows to visually evaluate the acoustic syndromes of TMJH, which is more objective and evident. Moreover, such a representation of sound constitutes 2D signal feature vector which can be fed into a convolutional neural net classifier capable of supporting automatic diagnosis [18].

The main goal of the research was to develop a novel diagnostic tool that has been verified on a group of patients suffering from TMD in order to describe the acoustic symptoms accompanying hypermobile temporomandibular joint and to compare them with physiological sounds and pathological clicks. In particular, the research was focused on patients who had no recognised sounds in RDC/TMD diagnosis.

Another aim was to provide a comparative analysis of the mentioned disorders from the digital sound processing standpoint. 

## 2. Materials and Methods

The study involved a group of patients with TMJ-connected aliments including temporomandibular joint hypermobility. The authors had the consent of Bioethics Committee number 1072.6120.71.2019. All the individuals expressed their written consent to participate in the examination and signed the consent forms prior to the study. During the procedures, the rules of good clinical practice were applied and the Helsinki Declaration was followed.

### 2.1. Standard Part of Examination

The criteria for inclusion were acoustic effects reported by patients with TMJ. The exclusion criteria were sounds from TMJ classified in the diagnosis according to RDC/TMD. For the participants who fulfilled the inclusion and exclusion criteria, Angle’s classes were additionally assessed and TMJ X-rays were performed in positions with both open and closed mouth. All the clinical procedures were performed by the first author, experienced in RDC/TMD examinations. The temporomandibular disorders were classified with the use of the Polish version of the RDC/TMD questionnaire [19]. The right and left sides of the body were considered separately [20,21]. Two axes of RDC/TMD were used.

### 2.2. Extened Examination Procedure

**Figure 1:**
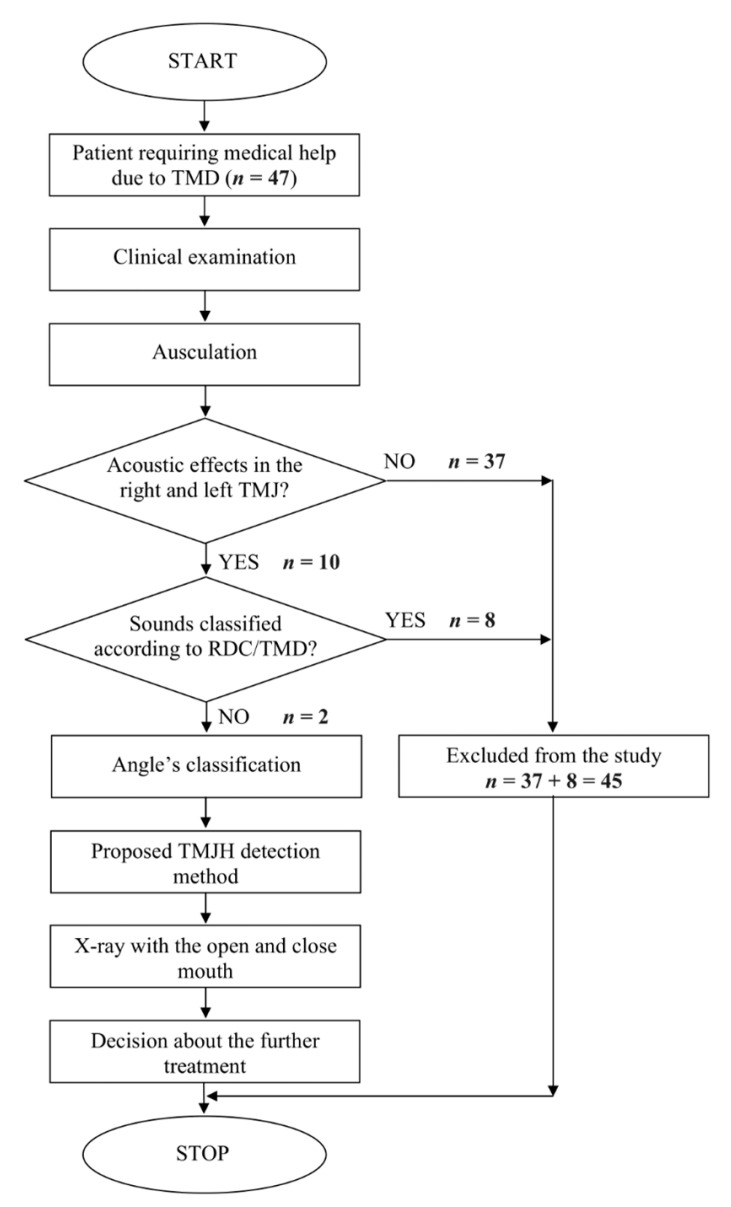
Flowchart of research procedure. TMJH: hypermobile temporomandibular joint; TMD: temporomandibular disorders; RDC/TMD: Research Diagnostic Criteria for Temporomandibular Disorders.

The overall examination and diagnostic procedure are visualised in the form of a flowchart (Figure 1).

Afterwards, each participant was auscultated by the same dentist, with two electronic stethoscopes (Littmann Model 3200 Manufacturer 3M Health Care, St. Paul, MN, USA). During this time, the patient was constantly opening and closing their mouth with non-standardised frequency, but in this period, there were approximately 5–8 repetitions. The auscultation was performed by applying the tip of the stethoscope to the facial skin in the preauricular areas to the right and left side simultaneously. In clinical practice, there are diagnoses in which the pathological sound is generated by only one of the joints. This phenomenon can result in mutual interference between the signals generated on both sides. The acoustic impedance of the bones and soft tissues along the route of propagation determine the frequencies obtained from the joint on the other side. Auscultation of patients with the mentioned symptoms is difficult to interpret. The comparison of signals acquired from both joints can provide diagnostic information about the origin of vibrations. To consider which signal is proper and which is artefact, calculation of the time delay between them is needed, which is the equivalent of measuring the phase shift between time synchronised signals. Such a technique was previously confirmed by us and the results have been published [22].

### 2.3. Signal Path and Processing 

Each Littmann 3200 device converts an analogue signal into the digital domain with 4000 Hz sampling frequency, which makes it possible to reproduce harmonic sound components up to 2000 Hz. Littmann 3200 can operate in three modes: standard bell, diaphragm (both are common in clinical practice in the case of pneumatic stethoscopes) and an additional extended mode. The bell mode amplifies sounds in the range of 20 Hz–1000 Hz with a stronger gain between 20 Hz–200 Hz. The diaphragm mode allows use of the full bandwidth (20 Hz–2000 Hz) with the range of 100 Hz−500 Hz emphasised. The extended mode was used in our research because of full bandwidth signal reproduction in the built-in pre-processing stage. The maximum amplification available in Littmann 3200 is 24× with 9 levels of regulation. In all the operating modes, the ambient noise reduction feature is enabled by default. A dedicated desktop application then enables the creation of a local database of patients, graphically visualises the signals and exports them in the audio file format for further processing. The assessment of acoustic symptoms was performed based on two TMJ sound signals recorded using the described stethoscope. Each measurement took around 15 s. It was not fatiguing for the patients and caused no discomfort. In addition, in some cases, the signals were recorded again, which made it possible to reach 30 s samples.

**Figure 2:**
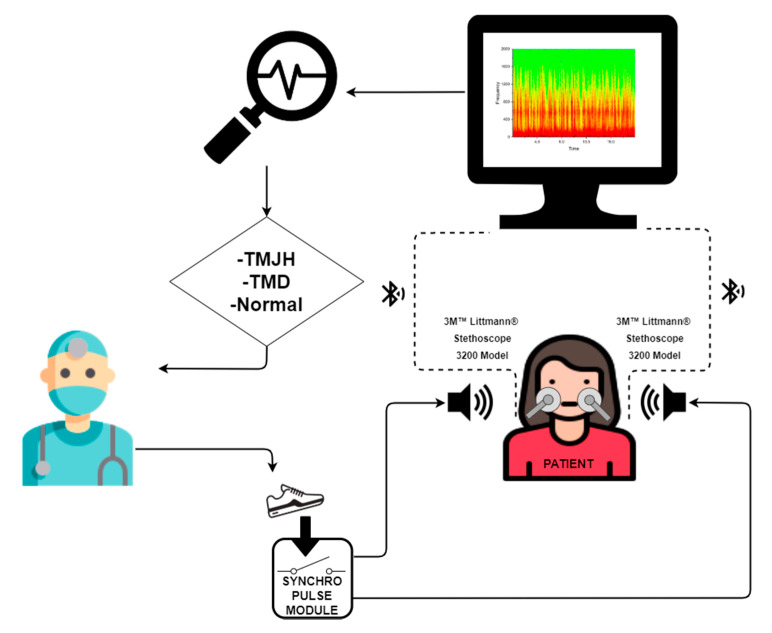
Visualisation of the entire examination procedure.

The measured sounds from electronic stethoscopes were sent to the computer to analyse the recorded signals. The whole examination procedure is presented in a simplified form in the diagram below (Figure 2).

The representation of the signal in the frequency domain was computed with the use of the Numpy library and the fast Fourier transform algorithm. Nevertheless, as it has already been stated, the global signal spectrum is not an efficient method of analysis of non-stationary signals. Therefore, in the course of the performed research, we provided short-time Fourier transform (STFT) of representative sounds calculated with the Matplotlib library. In order to achieve local spectral representation, we split the signal into atomic sections with the Blackman window of 512 samples length and 256 samples of overlapping. Spectrograms were presented in the linear time and frequency scale, while the magnitude of each component was represented in the logarithmic scale. The frequencies below 80 Hz were removed from spectrograms to emphasise higher harmonics that were responsible for the effect of clicking. The lower part of the bandwidth is persistent for the global spectral representation of each signal.

## 3. Results

The study group consisted of forty-seven volunteers, including twenty-nine women and eighteen men. The mean age of the participants was 32.46. The results of the RDC/TMD diagnoses are shown in Table 1.

Two responders (male and female aged 22 and 27, mean age 24.5 years) from the examined group fit the inclusion criteria—they had temporomandibular joint hypermobility—and this was 4% of the total respondents. The first patient was a female, age 27. The clinical examination showed Class II in the relation of Angle’s classification. The measurement between incisors during the maximum active opening was 44 mm and there were no muscle or joint pain complaints. The maximum passive opening was painless and amounted to 48 mm. In the examination carried out according to the RDC/TMD instruction, there were no noises during opening, closing, lateral or protrusive movements. In addition to the RDC/TMD questionnaire, the clinical examination showed a single sound like a thud at the end of the maximum opening in both joints. During palpation, neither muscle nor temporomandibular joint pain was reported. In Axis II, the patient assessed her general and oral health as good. She felt no pain in the face, ear or temple, but she observed a problem with sounds in both joints during wide opening and while eating hard food and yawning. This was the reason for seeking medical attention. With the examination with RDC/TMD, the result was without diagnosis. The next step consisted of a clinical examination with an electronic stethoscope.

The second patient was a 22-year-old male seeking dental care because of the sounds from TMJ during wide opening and closing. According to Angle’s classification, the occlusion was Class I. In Axis I RDC/TMD, the patient reported facial pain, especially muscle and joint pain on both sides of his face. He described the pain intensity as 3 in the scale of 0–3, 3 being the most painful. The vertical range between the upper and lower incisors during maximum opening without muscle or joint pain was 56 mm. The maximum active opening, with pain located in the muscles on both sides of the patient’s face, was 63 mm. The maximum passive opening was 65 mm, with pain in the muscles on both sides. In the examination following the instruction of RDC/TMD, no noises during opening, closing, lateral or protrusive movements were observed. However, there was a sound which was not classified in RDC/TMD. The acoustic effect was a sound like a thud during the end of the maximum mouth opening in both joints. During palpation, the pain in the muscles and the temporomandibular joint was reported: in the masseter, with the intensity of 2 on both sides and in the lateral pole of TMJ on both sides, with the intensity of 3. In Axis II, the patient assessed his general health as very good and his oral health as good. The individual had felt pain in the face, ear and temple in the previous month. He observed problems with sounds in both joints during wide opening and while eating hard food, yawning, and swallowing. He also reported ringing in his ears. In the examination with RDC/TMD, the diagnosis was Ia—myofascial pain and IIIa—arthralgia on both sides. At the end of the examination, the patient was auscultated with the electronic stethoscope.

**Figure 3:**
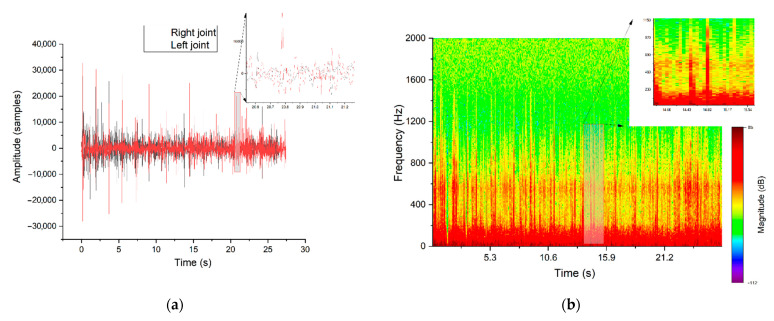
Signal traces recorded on both sides in the time domain (**a**) and the short–time Fourier transform (STFT) representation of a healthy TMJ (**b**)—representative parts of one jaw movement were magnified.

Both patients had hypermobility of the joints, which was confirmed by a functional X-ray of the temporomandibular joints. The X-ray examination exhibited the condylar translation. The signals recorded during auscultation were collected with the described setup based on a Littmann 3200 stethoscope and analysed using methods implemented in the Python 3 programming language (Figure 3).

**Figure 4:**
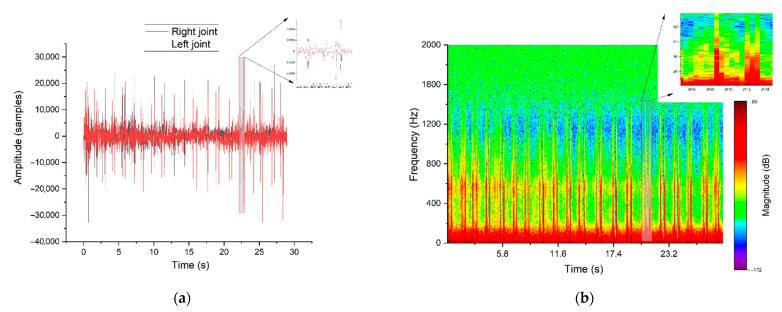
Signal traces recorded on both sides in time domain (**a**) and STFT representation of TMD manifested as clicks (**b**)—representative parts of one jaw movement were magnified.

**Figure 5:**
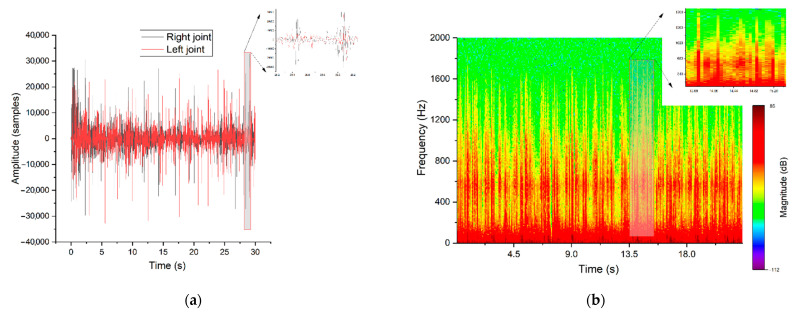
Signals traces recorded on both sides in the time domain (**a**) and STFT representation of TMJH manifested as a high-amplitude double-peak waveform (**b**)—representative parts of one jaw movement were magnified.

Using the graphical representation of the acquired sounds, it was possible to spot the visual morphological differences between particular clinical cases: healthy joint (Figure 3), TMD (clicking sounds) (Figure 4) and TMJH (Figure 5). Signals of healthy patients contain peaks in both representations that correspond to the final phase of jaw movement, and it is caused by physiological articular disc displacement. The spectrum is balanced for the entire duration of recording. 

Figure 4 depicts the original TMD sound with a visible significant peak waveform, which is typical for TMD. Another observation is that the amplitudes of vibrations corresponding to the joint movement between extreme positions are very low. Movements of TMJ structures of a healthy person are smooth and almost soundless according to the traditional auscultation criteria. As previously stated, the analysis of the non-stationary signals requires the observation of frequency changes as a function of time, which can be achieved through the application of time–frequency representation (spectrogram). As a result, characteristic frequencies can be distinguished in each section of the recorded joint movement. As mentioned, synchronised signal traces recorded on the left and right sides differ. Both TMJ and TMD generate sounds independently but a kind of crosstalk signal propagated by tissues from opposite sides should be taken into account; this is discussed in the literature [20]. Based on the obtained result in comparison with the physician’s observation and diagnosis, we can propose time–frequency representation as the most relevant. As highlighted in the figures, the diagnostic information of each recording is placed in a specific frequency and time range which directly corresponds with the movement stage.

Because of the small TMJH research group, which consisted of two cases only, no reliable statistical analysis was justified. However, based on a qualitive observation of the graphs, the TMJH can be characterised by periods of equilibrium in spectrograms, separated by high-amplitude peaks (Figure 5). However, crackles with diverse amplitude and noise are present during the whole recording of the TMJ sound diagnosed with clicks. Another typical feature of TMJH is represented by double vertical bars corresponding to the hypermobile clicks, which does not occur in pathological signals. As a result, the application of a spectrogram analysis provides the means to distinguish the hypermobility of TMJ from pathological clicks efficiently.

The high-amplitude peaks with a double-click form correspond to condylar dislocation out of the glenoid fossa and anteriosuperior to the articular eminence. The mandibular condyles, after wide opening, become trapped in front of the articular eminences. During the examination, an experienced dentist simultaneously auscultated the joints in the conventional way; as an additional diagnostic tool, the RDC/TMD questionnaire was applied. Based on this medical examination, pathologies were recognised. In the next step, the acquired signals were analysed using signal processing methods. Taking into account both aspects, specific signal compounds were determined. This part of our research is focused on finding distinguishable features of signals that can be implemented in a further step as an automatic tool for signal categorisation, which is the basis of the suggestions of diagnoses for the physician.

Figure 3, Figure 4 and Figure 5 represent the signal in time domain and its spectrogram for: patient with healthy TMJ, patient with TMJD and patient with TMJH, respectively. These cases can be recognised by spectrogram analysis, as all of them feature distinguishable visible effects. The colour shapes and patterns shown in the pictures and their mutual relations allow to differentiate the nature of the signals. Due to the signal conditioning process, the absolute intensity of the signal is not valuable. Time–frequency representation of the signal recorded for a healthy patient contains episodes of jaw activity visible as red bars, surrounded by significant noise caused by physiological displacement of joint surfaces. In the case of TMD the situation is similar; however, the signal-to-noise ratio during TMJ activity periods is higher in comparison to the healthy patient. Such an observation corresponds with the fact that the damaged TMJ generates clicks of high amplitude. The last picture (Figure 5) represents the results for the patient with diagnosed TMJH. In this case there are periods of equilibrium in the spectrogram, separated by high-amplitude peaks, which is caused by the double thud occurring when the patient suffers from joint hypermobility. The mentioned features allow to support diagnosis of healthy, damaged and hypermobile TMJ in a more objective manner than the standard auscultation.

## 4. Discussion

The result of 4% of patients with TMJH from the examined group confirms the worldwide statistical trend regarding TMJH cases. It is at a comparable level as what is stated in the literature. However, there are no standardised tools or criteria to determine if the patient suffers from TMJH. Thus, there is no single reference concerning TMJH prevalence. However, the obtained results can be confronted with the studies described below.

The hypermobile joint syndrome concerns between 5% and 10% of populations and is more prevalent among women [23]. The research of Chang et al. noticed that not everyone affected by the hypermobile joint syndrome has TMD. Approximately 9.52% of the examined group have a TMD and 90.48% are healthy [24]. The occurrence of 21–42% of hypermobility depending on the criteria and score of the Beninhton scale was observed by Collinge et al. [25].

The conclusion of higher prevalence of hypermobility in a group of women was confirmed by Hirsch et al. The researchers described that in the group of people who suffer from hypermobility (2.8–14.8% of examined patients), GJH was found in 6.9% of the subjects. The discrepancy of the frequency of occurrence was caused by diagnosis [26]. The hypermobility examined with the Beighton scale can be a single diagnosis or it can exist as a part of the diagnostic criteria of Marfan and Ehlers–Danlos syndromes [27]. It is difficult to unequivocally state what is the reality of the occurrence of hypermobility because scientists use different criteria and scales to make a diagnosis. In addition, different populations are studied, the age of the patients is heterogeneous, and there are various comorbidities. An important issue is also the unrecognisability of hypermobility. Kirk et al. noticed that hypermobility is widespread, but most symptoms are mild and ambiguous which means that the diagnoses are not reliable [7]. The rare occurrence confirms the percentage obtained during our research.

A relevant fact is that in the standard RDC/TMD classification, there is no diagnosis for subjects who suffer from the sound referred to as a thud in the final phase of the mandibular opening movement [19,20,21]. Moreover, currently only further clinical examination and additional medical investigation such as X-ray, ultrasonography or MRI can confirm the TMJH diagnosis. Thus, it should be stressed that our methodology does not require such complex procedures. Many advantages of using the stethoscope for the purposes of the TMJ diagnosis can be pointed out. First of all, electronic auscultation is an examination that can be performed during a patient’s visit and immediately analysed thanks to measurement data visualisation on the computer screen. Furthermore, recorded acoustic signals and their representations in the frequency and time domain are characteristic for each specific sound-correlated dysfunction of TMJ. This provides a way of detecting a particular TMJ disorder. A stethoscope can be used in certain cases when X-rays are contraindicated (e.g., in the case of pregnancy).

TMJH predisposes to TMD, which is why some patients require the use of occlusive splints, muscle relaxation or speech therapy exercises [28]. This is one of the reasons why dentists need repeatable solutions to control therapies. Bakalczuk et al. used an advanced solution, BioJVA, for this purpose [29]. Another advantage of our method is the possibility of storing the measured data. During the treatment procedure, the dentist can auscultate the patient, record the data, and compare the current results to the results from the previous appointment. It is possible to monitor ongoing therapy. Loster et al. observed that more objective instruments are needed for the assessment and treatment of patients with TMD [30].

Using our above-mentioned method, it seems easier to observe a worsening clinical situation leading, for example, to arthritis. In the case of insufficient physician experience in this field, it is possible to share the data and consult with a specialist. This telemedical functionality benefits the quality and availability of treatment. Jasim et al. used a stethoscope to diagnose the influence of third molars on the appearance of sounds in temporomandibular joints [31]. Krohn et al. used an intraoral sensor for monitoring patients with splint therapy [32]. Many researchers have tried to combine technical solutions with physical examination and these attempts have led to new conclusions.

Al-Huraishi et al. examined the knowledge of TMD among regular dentists, in comparison to specialists, on a validated questionnaire [33]. The researchers observed a low level of knowledge in this area among new graduates. The examination of TMJ is especially difficult for young people. The combination of technical solutions and telemedicine can increase the accuracy of diagnoses. This was one of the reasons why our research tried to support dentists.

Within the research, the examination of the TMJ was performed by recording the sound accompanying free movement of the joint in all accessible ranges of the movement. This is a significant difference and advantage in comparison with other X-ray-based medical imaging methods, which only provide information regarding geometrical characteristics of the joint or the internal bone structure. Standard imaging methods do not deliver sufficient information concerning the influence of low-density tissues such as muscles or ligaments, not to mention synovial fluid or microscopic phenomena occurring on the moving joint surfaces (grease layer, local Hertz stress, etc.). Such methods have limited applications in the TMJ diagnostics due to the fact that the measurement of geometry is usually performed in the static state, with no jaw motion. In addition, the proposed method enables acquisition of the acoustic signals of the TMJ that take place during the functional activity of the joint. Such conditions allow investigation of the influence of all the sources of joint dysfunction, such as the degeneration of interarticular surfaces and synovial fluid or excessive muscle tension.

The presented analysis of acoustical signals of TMJ activity was performed in the time–frequency domain using the STFT. Based on the obtained results, it can be stated that, thanks to the application of the time–frequency analysis, it is relatively easy to distinguish the hypermobility of TMJ from pathological clicks. It should be emphasised that such an analysis could be performed immediately after the patient examination. Therefore, auscultation can be complemented with the observation of signals displayed in the form of spectrograms.

The main aim of the conducted research is the development of a generalised method of testing and identifying TMJH that can be used without major restrictions concerning equipment availability, specific personnel qualifications or the use of individually adapted computational algorithms and recognition procedures. The presented research has already revealed some limitations and inconveniences of the proposed method application. Significant limitations are related to the common disadvantages of the stethoscope method, where disturbances are generated during random movements of the stethoscope head along the skin surface. Moreover, for the TMJH patients, the characteristic sounds occur in the case of the wide opening of the mouth, while the abduction movement across a small range is soundless. Sensitivity of the TMHJ identification algorithms is also limited, its efficiency decreases along with the increase in noise despite the used anti-interference and noise filters. In practice, it is necessary to gain experience to avoid generating unnecessary noise and rubbing the stethoscope membrane against the skin. The third group of limitations at this stage of research results from the lack of access to a statistically representative group with confirmed cases of TMJH diagnosed with other medical imaging methods, which does not allow for a detailed analysis of the correlation between the sensitivity of the method and the size of the anatomical changes confirmed by X-ray.

## 5. Conclusions

Based on the performed research, we observed that hypermobile joints are rarely encountered among TMD patients. In our study group, 4% of subjects were diagnosed with TMJ sounds like a thud. A comparable incidence level of this ailment is stated in the literature which was mentioned in the Discussion section. However, this prevalence of problematic diagnosis indicates the practical usefulness of the presented method in everyday clinical practice. Due to the uncommon occurrence of TMJH in the whole population, the study should be continued to increase the number of clinically confirmed TMJH cases. The obtained result will contribute to expanding dental knowledge regarding TMJ.

For further analysis, recordings of TMJ sounds were obtained, including those of patients with TMJH. However, due to the small number of the TMJH patients, it was not possible to create a comprehensive, reference database of these sounds. These studies are being continued, so the development of such a database is planned, as is its delivery to readers and other researchers. In the Results section, spectrograms of the TMJ sound from subjects suffering from joint clicking and patients diagnosed with TMJH were presented. The conducted investigation revealed that these two cases with TMJH can be distinguished based on the characteristic time–frequency features. Such a diagnosis is usually not feasible in clinical conditions since both acoustic signals sound similar and are impossible to differentiate by the human ear. Unfortunately, many specialists in the field of dentistry, even prosthodontic specialists, have problems with the diagnosis and treatment of sounds from temporomandibular joints. Our research tries to present a cheap, quick and available solution to help the dentist in the recognition of sounds from joints. Despite the low number of people with temporomandibular joint hypermobility (confirmed in approximately 4% of patients), it is worth considering. It seems realistic that TMJH is undiagnosed in many patients, even the criteria for diagnosing hypermobility are not standardised. For this reason, we should try to detect people with such a diagnosis to help patients in the case of, for example, pain symptoms. In some extreme cases, the early detection and treatment of TMJH can reduce the formation of long-term, more severe joint pathologies, such as articular capsule stretching or recurrent mandibula blocking in the open position. Even in the case of rare diagnoses, the patients’ quality of life is worth improving when possible. We believe that our idea will contribute to such a goal.

## Figures and Tables

**Table 1:** RDC/TMD Axis I diagnosis in the examined group of a total of 47 patients.

RDC/TMD Axis I Group Diagnoses	Participants	Fraction
I—muscle disorders	7	14.89%
II—disc displacements	15	31.91%
III—arthralgia, osteoarthrosis	11	23.40%
Total with RDC/TMD Axis I diagnoses	20	42.55%
Total without Axis I RDC/TMD diagnoses	27	57.45%

RDC/TMD: research diagnostic criteria for temporomandibular disorders.

## Data Availability

The data presented in this study are available on motivated request to the corresponding author.
